# MUC1 (CA27.29) before and after Chemotherapy and Prognosis in High-Risk Early Breast Cancer Patients

**DOI:** 10.3390/cancers14071721

**Published:** 2022-03-28

**Authors:** Hanna Huebner, Lothar Häberle, Volkmar Müller, Iris Schrader, Ralf Lorenz, Helmut Forstbauer, Visnja Fink, Fabienne Schochter, Inga Bekes, Sven Mahner, Julia Jückstock, Naiba Nabieva, Andreas Schneeweiss, Hans Tesch, Sara Y. Brucker, Jens-Uwe Blohmer, Tanja N. Fehm, Georg Heinrich, Mahdi Rezai, Matthias W. Beckmann, Peter A. Fasching, Wolfgang Janni, Brigitte Rack

**Affiliations:** 1 Department of Gynecology and Obstetrics, Comprehensive Cancer Center EMN, Erlangen University Hospital, Friedrich-Alexander-Universität Erlangen-Nürnberg (FAU), 91054 Erlangen, Germany; hanna.huebner@uk-erlangen.de (H.H.); lothar.haeberle@uk-erlangen.de (L.H.); naiba.nabieva@fau.de (N.N.); matthias.beckmann@uk-erlangen.de (M.W.B.); 2 Biostatistics Unit, Department of Gynecology and Obstetrics, Erlangen University Hospital, Friedrich-Alexander-Universität Erlangen-Nürnberg (FAU), 91054 Erlangen, Germany; 3 Department of Gynecology, University of Hamburg-Eppendorf, 20251 Hamburg, Germany; vmueller@uke.uni-hamburg.de; 4 Breast Center, Diakovere Henriettenstift, 30171 Hannover, Germany; iris.schrader@me.com; 5 Gynecologic Practice Dres Lorenz, Hecker, Wesche, 38100 Braunschweig, Germany; lorenz@lorenz-hecker-wesche.de; 6 Hemato-Oncological Practice Dres Forstbauer and Ziske, 53840 Troisdorf, Germany; forstbauer@onkologie-rheinsieg.de; 7 Department of Gynecology and Obstetrics, Ulm University Hospital, 89081 Ulm, Germany; visnja.fink@uniklinik-ulm.de (V.F.); fabienne.schochter@uniklinik-ulm.de (F.S.); wolfgang.janni@uniklinik-ulm.de (W.J.); brigitte.rack@uniklinik-ulm.de (B.R.); 8 Clinic for Medical Oncology and Hematology, Cantonal Hospital St. Gallen, 9000 St. Gallen, Switzerland; inga.bekes@kssg.ch; 9 Department of Obstetrics and Gynecology, University Hospital, Ludwig-Maximilians-Universität München (LMU), 80337 Munich, Germany; sven.mahner@med.uni-muenchen.de (S.M.); julia.jueckstock@med.uni-muenchen.de (J.J.); 10 National Center for Tumor Diseases, Heidelberg University Hospital, 69120 Heidelberg, Germany; andreas.schneeweiss@med.uni-heidelberg.de; 11 Department of Gynecology and Obstetrics, Heidelberg University Hospital, 69120 Heidelberg, Germany; 12 Department of Oncology, Onkologie Bethanien, 60389 Frankfurt, Germany; hans.tesch@chop-studien.de; 13 Department of Gynecology and Obstetrics, Tübingen University Hospital, 72076 Tübingen, Germany; sara.brucker@med.uni-tuebingen.de; 14 Department of Gynecology, Breast Center, Charité-Universitätsmedizin, 10117 Berlin, Germany; jens.blohmer@charite.de; 15 Department of Gynecology and Obstetrics, Düsseldorf University Hospital, Heinrich Heine University, 40225 Düsseldorf, Germany; tanja.fehm@med.uni-duesseldorf.de; 16 Department of Gynecologic Oncology, Schwerpunktpraxis für Gynäkologische Onkologie, 40235 Fürstenwalde, Germany; praxis.heinrich@t-online.de; 17 Department of Breast Diseases, Breast Center of Duisburg, Sant Ana Hospitat, 47259 Duisburg, Germany; mahdi@rezai.org; 18 Department of Medicine, Division of Hematology/Oncology, David Geffen School of Medicine, University of California at Los Angeles, Los Angeles, CA 90095, USA

**Keywords:** early breast cancer, tumor marker, chemotherapy, anthracycline, taxane, MUC1, CA27.29, CA15-3

## Abstract

**Simple Summary:**

CA27.29 (MUC1) is a well described biomarker for prediction of prognosis and treatment efficacy. CA27.29 is mainly evaluated in the preoperative setting. However, testing of postoperative levels and additional assessment after chemotherapy might be more informative for analyzing the usefulness of CA27.29 in relation to the efficacy of chemotherapy. Thus, both pre- and post-chemotherapy values were assessed from patients enrolled in the breast cancer SUCCESS-A trial. Pre-chemotherapy assessment was associated with disease-free survival. It had no prognostic value in node-negative patients, but there was a clear association in node-positive patients. Furthermore, it was shown that post-chemotherapy CA27.29 assessment did not add any prognostic value, either on its own or in addition to pre-chemotherapy assessment. In conclusion, this indicates that pre- and post-chemotherapy values do not provide additional information. However, pre-chemotherapy CA27.29 could be a suitable tool to identify a group with unfavorable prognosis among node-positive patients.

**Abstract:**

Soluble MUC1 has been discussed as a biomarker for predicting prognosis, treatment efficacy, and monitoring disease activity in breast cancer (BC) patients. Most studies in adjuvant settings have used preoperative assessment. This study, part of the SUCCESS-A trial (NCT02181101), assessed the prognostic value of soluble MUC1 before and after standard adjuvant chemotherapy. Patients with high-risk BC were treated within the SUCCESS-A trial with either three cycles of 5-fluorouracil, epirubicin, and cyclophosphamide followed by three cycles of docetaxel or three cycles of FEC followed by three cycles of docetaxel and gemcitabine. Cox regression analyses were performed to investigate the prognostic value of CA27.29 before and after chemotherapy relative to disease-free survival (DFS), along with established BC prognostic factors such as age, body mass index, tumor size, nodal status, estrogen receptor, progesterone receptor, HER2 status, and grading. Pre-chemotherapy and post-chemotherapy CA27.29 assessments were available for 2687 patients of 3754 randomized patients. Pre-chemotherapy CA27.29 assessment was associated with DFS in addition to established prognostic factors. It had no prognostic value in node-negative patients, but there was a clear association in node-positive patients. Post-chemotherapy CA27.29 assessment did not add any prognostic value, either on its own or in addition to pre-chemotherapy CA27.29 assessment.

## 1. Introduction

Biomarkers that help to monitor the efficacy of treatment may be extremely useful for predicting prognosis, guiding the therapy and assessing the early response to it [1]. There has been growing interest in the extent to which tumor markers may be of value for predicting the prognosis and treatment response in individual patients and for monitoring therapy.

One of these biomarkers is MUC1, which is overexpressed in many malignancies, particularly in breast cancer (BC) [2,3]. It comprises two subunits, a transmembrane (MUC1-C) and an extracellular subunit (MUC1-N). MUC1-C has been described as having oncogenic functions, interacting with receptor tyrosine kinases and activating several pathways including the PI3K-, MAPK-, NFκB-, and Wnt/β catenin pathways [3]. 

MUC1-N can be shed, is soluble in serum, and can be measured with antibodies directed against epitopes of this subunit. The shed and soluble subunit is often referred to using the name of the antigen (CA27.29 or CA15-3)—i.e., the target of the analytic assay for the measurement of MUC1 levels [2]. 

MUC1 is a prognostic marker in both early [4,5,6,7,8,9,10,11,12,13,14,15,16,17,18] and advanced BC [19,20]. In most studies in patients with non-metastatic BC, blood for MUC1 assessment has been obtained preoperatively. Clear associations were found between tumor size and the soluble MUC1-level. While large studies have shown that soluble MUC1 is a predictor of prognosis for both early and advanced tumor stages independently of the tumor stage [7,9,15], it may be hypothesized that the close association between tumor stage and MUC1 is due to the fact that MUC1 levels mirror the tumor burden in BC patients. However, testing of postoperative levels and additional assessment after chemotherapy might be more informative for analyzing the usefulness of MUC1 in relation to the efficacy of chemotherapy and other biological effects, such as its role in early systemic tumor spread.

The aim of this study was therefore to assess the association with disease-free survival and MUC1 as measured using a CA27.29 assay before and after adjuvant chemotherapy, taking into account established prognostic factors.

## 2. Materials and Methods

### 2.1. Study Design

In the SUCCESS-A open-label phase 3 trial, patients were randomly assigned at a ratio of 1:1 to either an anthracycline/taxane-based chemotherapy or to this chemotherapy regimen plus gemcitabine [21]. Further treatment specifications are provided in the Supplementary methods. The SUCCESS-A study was conducted as an investigator-initiated and led trial in Germany approved by all the ethics committees responsible and was conducted in accordance with the Declaration of Helsinki. All of the patients provided written informed consent before entering the study.

### 2.2. Participants

Patients were eligible if they were aged 18 or older and had a diagnosis of early, non-metastasized, high-risk invasive BC, defined by tumor stage, tumor grade, hormone receptor status or age. The full inclusion/exclusion criteria are provided in Additional Table 1.

### 2.3. End Points, Follow-Up, and Data Capture

Disease-free survival was defined as the time from the last chemotherapy administration to the earliest date of disease progression (distant metastasis, local recurrence, death from any cause) or the date of censoring. Patients who were lost to follow-up before the maximum observation time of 5.5 years or were disease-free after the maximum observation time were censored at the last date on which they were known to be disease-free or at the maximum observation time. The maximum observation period of 5.5 years consists of six months of chemotherapy and (up to) five years of follow-up thereafter.

For survival and recurrence assessment, the patients were followed at the study sites at 3-month intervals for the first three years and every six months thereafter. The follow-up included clinical examinations (at each visit), mammography (every six months), and symptom-driven examinations if necessary. High quality of the data was ensured by electronic data management, including automated plausibility checks and regular monitoring visits to the study site by an independent clinical research organization (Alcedis GmbH, Giessen, Germany) and a data monitoring committee (DMC).

### 2.4. Assessment of Soluble MUC1 with the CA27.29 Assay

Laboratory analysis was performed centrally in the Department of Gynecology and Obstetrics at Munich University Hospital [22]. Approximately 10 mL of peripheral blood was drawn by peripheral vein puncture in standard serum tubes and centrifuged (10 min, 2000× *g*, room temperature) within 24–72 h following the collection time to remove clots. The serum was immediately transferred to an immunoreaction cup from the ST AIA-Pack 27.29 series (Tosoh Bioscience, Tessenderlo, Belgium) for further analysis. The CA27.29 serum concentration was measured using the AIA-600 II automated enzyme immunoassay system (Tosoh Bioscience, Tessenderlo, Belgium) in accordance with the manufacturer’s instructions. In brief, serum samples were combined with a diluent (1:20) and were transferred to an immunoreaction cup from the ST-AIA-Pack 27.29 series. CA27.29 was immobilized using magnetic beads conjugated to antibodies. Enzyme-labeled antibodies attached to a different epitope were then bound to the CA27.29 antigen in a sandwich manner. The samples were then incubated at 37 °C, followed by a washing step to remove any unbound antibody. The fluorogenic substrate 4-methylumbelliferyl phosphate was added to the test cup, and enzyme activity was measured on the basis of the amount of fluorescence.

### 2.5. Statistical Methods

The primary objective was to study whether information about CA27.29 before and after chemotherapy improves the ability to predict disease-free survival for each patient, in addition to other well-known predictors. For this purpose, Cox regression analyses were performed as described in the Supplementary methods [23,24,25,26,27,28]. In brief, a Cox regression model for commonly known predictors was compared with a further model containing those variables and additionally CA27.29 before and after chemotherapy and all relevant interaction terms for those variables (“full model”) using the likelihood ratio test (LRT). In case of significance, the full model was tested for the relevance of the included interaction terms by comparing the full model with a model without interaction terms using a second LRT. In the case of significance, a variable selection procedure was carried out to identify relevant interaction terms (“final model”). Hazard ratios (HRs) and survival rates were estimated using the final model. Furthermore, the predictive ability of CA27.29 before and after chemotherapy was compared.

All of the tests were two-sided, and a *p* value of <0.05 was regarded as statistically significant. Calculations were carried out using R statistical software (V3.0.1, 2013, The R Foundation for Statistical Computing, Vienna, Austria). Remark criteria were used to report tumor marker data [29].

## 3. Results

### 3.1. Patient Characteristics

**Figure 1:**
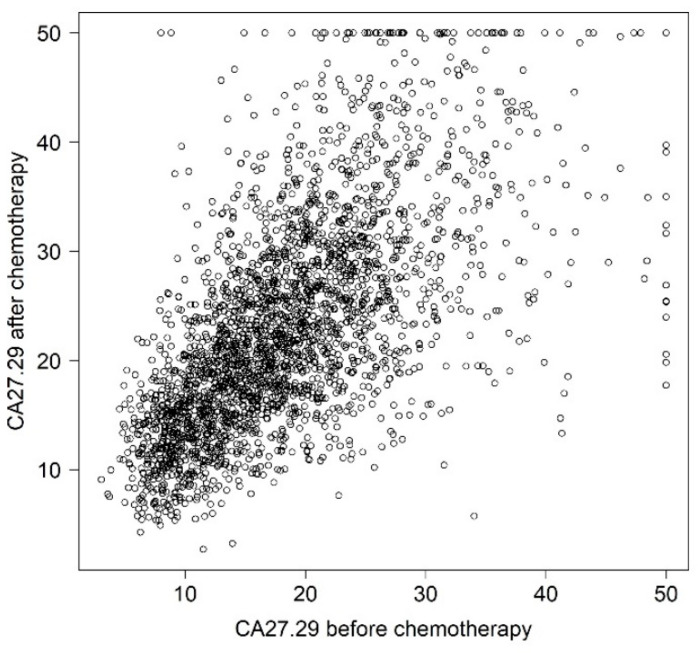
CA27.29 before chemotherapy versus CA27.29 after chemotherapy. Outliers were truncated at 50 U/mL.

In total, 3754 patients were randomly assigned in the SUCCESS-A study. For inclusion in the present analysis, patients were required to have serum samples for CA27.29 analysis at the time of study inclusion and after chemotherapy (*n* = 2687). Complete information on all variables as listed in Table 1 was available for 98.2% of these patients. The percentage of values missing for each variable was less than 0.5%, with the exception of HER2 (1.5%). The characteristics of the patients are shown in Table 1. CA27.29 values before and after chemotherapy were strongly correlated (Figure 1).

### 3.2. Prediction of Disease-Free Survival

CA27.29 was significantly associated with disease-free survival additionally to other predictors (*p* < 0.000001, first LRT). The effect of CA27.29 on survival differed between patient subgroups (*p* < 0.001, second LRT). The variable selection process resulted in a final Cox regression model that included, besides the predictors of the reduced model, the interactions of CA27.29 before chemotherapy relative to lymph node status (pN) and tumor size (pT). CA27.29 before chemotherapy was nonlinearly associated with survival, with two degrees of freedom, whereas CA27.29 after chemotherapy was best described as a linear predictor.

The expected probability of 5-year survival was calculated for each patient using the final model. Many patients were found to have a high probability of living free of disease for at least five years, whereas only a few patients had a low probability (Figure S1). The mean and median probabilities were 85.9% and 88.6%, respectively. One quarter of all patients had a likelihood less than 82.6%, and one quarter had a likelihood greater than 92.5%.

**Figure 2:**
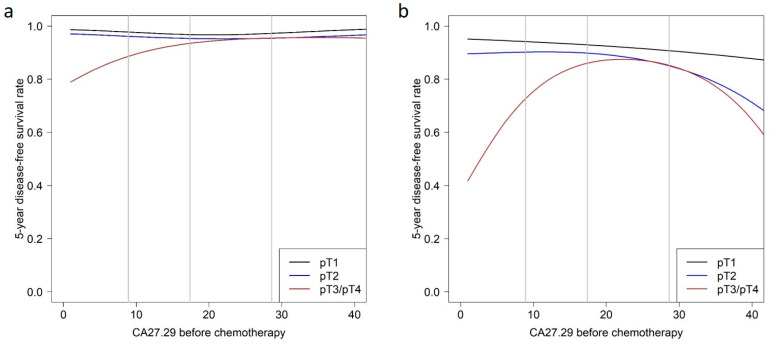
The 5-year disease-free survival rate as a function of CA27.29 before chemotherapy relative to tumor size for (**a**) patients with negative lymph-node status and (**b**) patients with positive lymph-node status. Vertical gray lines indicate the 25th, 50th, and 75th percentiles for CA27.29 before chemotherapy.

Patient subgroups were defined based on the two predictors pN and pT. pN0 is defined as no cancer cells in any nearby nodes and pN+ as the presence of cancer cells in lymph nodes. pT1 means that the tumor is 2 cm across or less, pT2 means that the tumor size is more than 2 cm but no more than 5 cm, and pT3/4 means that the tumor is bigger than 5 cm or of any size growing into the chest wall or skin. No association of CA27.29 before chemotherapy with survival was observed in patients with lymph node-negative tumors (Table 2 and Figure 2a).

**Figure 3:**
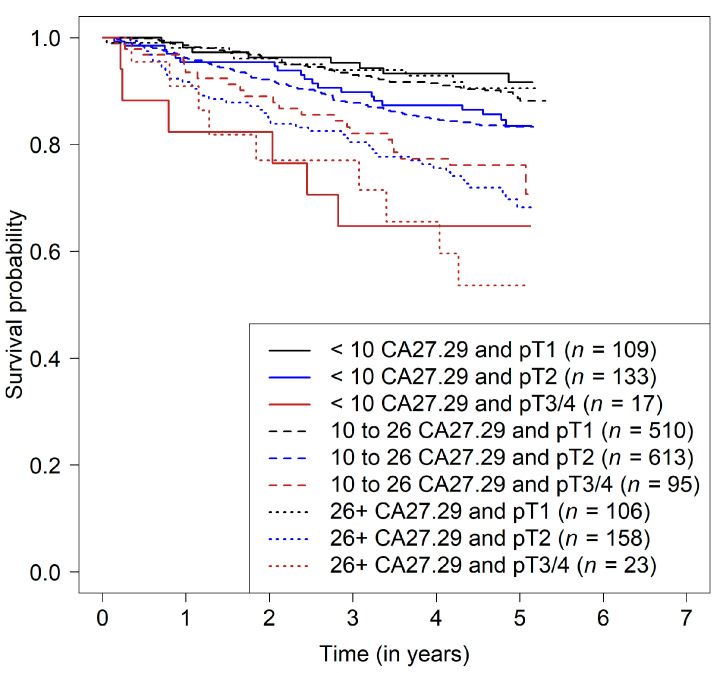
Kaplan–Meier curves for disease-free survival relative to pT and CA27.29 before chemotherapy grouped into three almost equal categories (“low”, <10; “intermediate,” from 10 to 26; “high”, 26 or more) in lymph node-positive patients.

In patients with lymph node-positive tumors, CA27.29 only showed a minor association with survival in patients with pT1 tumors. For each CA27.29 value before chemotherapy, the survival rate for patients with pT2 tumors was lower than that of patients with pT1 tumors with the same CA27.29 value. In patients with pT2 tumors, survival rates were constantly high when CA27.29 was below the median, but the survival rates decreased with increasing CA27.29 values. The survival prognosis in patients with large tumors (pT3 or pT4) improved with increasing CA27.29 values, but only up to values of 25 U/mL. Patients with CA27.29 values higher than 25 U/mL had a prognosis similar to that in patients with pT2 tumors—the higher the CA27.29 value, the poorer the prognosis. Moreover, the survival rates of patients with pT2 tumors were similar to those of patients with pT3/4 tumors in this range of above-average CA27.29 (Table 2 and Figure 2b and Figure 3).

No association between CA27.29 after chemotherapy and the disease-free survival was found. The adjusted HR per 10 U/mL increase of CA27.29 was 0.95 (95% CI, 0.83–1.10).

In summary, a “high-risk patient” is a patient with a lymph-node positive tumor with at least pT2 and high CA27.29 values before chemotherapy or pT3/4 and low or high but not intermediate CA27.29 values before chemotherapy. The survival prognosis is further increased or decreased, respectively, by histology, grading, ER, PR, and HER status. For instance, a patient with a tumor classified as T2, G3, ER+, PR+, and HER− is predicted to have a 5-year disease-free survival with a likelihood of 85% if the preoperative CA27.29 level was low and a likelihood of 75% if the preoperative CA27.29 was high. Table 3 shows the predicted 5-year survival rates obtained from the final model.

Both the full and final models performed better than the basic model without CA27.29 predictors with regard to distinguishing between patients with and without events up to two and five years of follow-up, respectively (Supplementary Table S2, cross-validated AUC). The full model, however, performed better than the final model; the difference in the cross-validated AUC was 0.041 at two years and 0.06 at five years, implying a certain joint influence of predictors that are poorly predictive on their own. A comparison between the apparent and cross-validated AUC shows that the final model was less overfitted than the full model. The addition of CA27.29 before chemotherapy to the basic model improved prediction, whereas the addition of CA27.29 after chemotherapy did not, confirming the results of the main analysis.

## 4. Discussion

This study showed that CA27.29 after surgery but before chemotherapy provides prognostic information additional to that available with commonly known prognostic factors. CA27.29 after chemotherapy did not add any prognostic information in addition to commonly known prognostic factors and CA27.29 before chemotherapy. The impact of CA27.29 differed relative to tumor size and nodal status—mainly confining the association of CA27.29 and prognosis to node-positive patients.

In this setting, serial use of CA27.29 did not increase its usefulness as a biomarker. However, there have been several reports in which it was not a single, static soluble MUC1 value that was used for prognostic or predictive purposes, but rather serial measurements with adjustment of later values by the earlier ones [19,30,31,32,33,34,35,36,37,38,39,40]. There are several potential reasons why the two values in the present study were not superior to the one before chemotherapy alone. Firstly, the two assessments were highly correlated (Figure 1). Secondly, the values after chemotherapy may reflect a baseline value for MUC1 rather than an activity marker for the disease [30], assuming that at the second time point, the time interval is long enough to exclude any influence of the primary disease on the MUC1 value, and the likelihood of recurrence is rather low. The prognostic information provided by CA27.29 after chemotherapy may therefore be rather low.

Before chemotherapy, two interactions were observed: one between tumor size and CA27.29, and one between nodal status and CA27.29. Patients with a negative nodal status had an excellent prognosis. However, no association between CA27.29 and the prognosis was observed. This might be due to general better prognosis of node-negative BC patients resulting in less impact of CA27.29 levels [41,42]. 

An effect of CA27.29 on the prognosis was seen in node-positive patients, with the exception of patients with advanced tumor stages (pT3/4). In the latter, low CA27.29 values were associated with an unfavorable prognosis. Interactions between molecular subtypes were not analyzed since the assumptions for the proportional hazard function were not met and there was a strong correlation between ER and PR. Several larger studies have reported associations between tumor size, nodal status, and MUC1 [7,9,12,17]. Patients with larger tumors and positive lymph-node status had higher preoperative MUC1 values. Postoperative values were assessed in only one of these studies [15], but no association between tumor size, nodal status and CA15-3 was reported. In the present study, there was an association between tumor size and postoperative/before chemotherapy CA27.29 values, but not with nodal status, a finding that was also reported by Rack et al. [43].

Only one study has investigated the association of postoperative MUC1 assessments in relation to other prognostic factors (tumor size, lymph-node status, histological grading, and hormone receptor status) [15]. Carcinoembryonic antigen as well as CA15-3 was included but did not have a prognostic effect in the multivariable model. The study also found that postoperative values differ from preoperative ones [15]. Approximately 18% of all patients had a decrease in CA15-3 values of more than 33%. The decrease was even more prominent in node-positive patients [15]. This might be an indication that MUC1 plays a different role in node-positive patients than in node-negative ones, but it might also be due to the fact that node-negative patients do not have elevated MUC1 levels initially and can therefore not achieve a decrease after surgery that easily.

MUC1 has been widely discussed as a prognostic biomarker and potential target for anticancer treatment [44,45]. In addition to this, however, MUC1 has been linked to immune regulatory mechanisms such as protection from infections, regulation of inflammatory response and, in particular, its ability to function as a T cell regulator and checkpoint molecule [46,47,48,49]. With respect to the latter, co-stimulatory and co-inhibitory abilities are discussed, which might influence the activity of regulatory T cells, but also CD4+ or CD8+ cells in general [47,48]. Thus, the association of low CA27.29 values and poor prognosis of node-positive patients with advanced tumor stage (pT3/4) might be due to the loss of the MUC1-regulated immune response. A lack of co-stimulatory and co-inhibitory MUC1 activities might particularly impact patients with advanced tumor stage and cancer-affected lymph nodes. Thus, for future studies, it could be of interest to evaluate the proliferation, differentiation, and activation of circulating immune cells in addition to CA27.29 values [49]. 

MUC1 has been prioritized as a promising target for anticancer therapies [44,45]. Vaccination strategies appear to be of particular interest, as MUC1 is overexpressed in BC and other cancer histologies and has been reported to have a high level of immunogenicity [45,50]. The present study shows that node-negative patients may not be ideal for an anti-MUC1 therapeutic study. Patients should potentially be node-positive with high MUC1 serum levels to derive benefit.

This study has several strengths and weaknesses. It is the first large-scale study in which samples have been analyzed in a multicenter prospective phase III chemotherapy investigation. CA27.29 was assessed centrally, ensuring high-quality data and standardized analytic methods. It therefore appears to be justifiable to generalize the results, although the study was restricted to a high-risk group undergoing chemotherapy. While the overall sample size was reasonably high with more than 2600 patients, the unfavorable prognostic effect in patients with pT3/4 and low CA27.29 may be imprecise due the small sample size for that specific patient group.

## 5. Conclusions

In conclusion, this study did not show additional prognostic value for serial CA27.29 assessment before and after chemotherapy alongside commonly used prognostic factors and postoperative CA27.29 measurements. In combination with tumor size and nodal status, as well as other prognostic factors, however, serial CA27.29 measurements can identify patients with an unfavorable prognosis. This patient population may be suitable for further anti-MUC1 treatment after standard therapies.

## Figures and Tables

**Table 1:** Patient Characteristics.

Characteristic	Mean or Count	SD or %
Age	53.0	10.5
BMI	26.2	5.0
CA27.29		
Before chemotherapy	18.4	8.1
After chemotherapy	23.1	9.8
pT		
pT1	1106	41.2
pT2	1411	52.5
pT3	137	5.1
pT4	33	1.2
pN		
pN+	1764	65.6
pN0	923	34.4
Histology		
Ductal	2202	82.0
Lobular	302	11.2
Other	183	6.8
Grading		
G1	118	4.4
G2	1304	48.5
G3	1265	47.1
ER		
ER−	922	34.3
ER+	1765	65.7
PR		
PR−	1091	40.6
PR+	1596	59.4
HER2/neu		
HER2−	2038	75.8
HER2+	649	24.2

BMI, body mass index; ER, estrogen receptor; PR, progesterone receptor; SD, standard deviation.

**Table 2:** Cox Regression Analysis of Disease-Free Survival, * Showing Subgroup-Specific ^†^ and Adjusted ^‡^ Hazard Ratios for CA27.29 before Chemotherapy, with 95% Confidence Intervals.

Patient Subgroup	Hazard Ratio (95% Confidence Intervals) for CA27.29 before Chemotherapy		
	Medium vs. Low	High vs. Medium	High vs. Low
pN0 and pT1	1.42 (0.77, 2.60)	0.85 (0.52, 1.39)	1.20 (0.52, 2.77)
pN0 and pT2	1.20 (0.70, 2.07)	0.97 (0.64, 1.46)	1.17 (0.55, 2.45)
pN0 and pT3/4	0.55 (0.26, 1.17)	0.68 (0.41, 1.12)	0.38 (0.13, 1.08)
pN+ and pT1	1.23 (0.78, 1.95)	1.34 (0.94, 1.90)	1.64 (0.86, 3.14)
pN+ and pT2	1.04 (0.75, 1.45)	1.52 (1.25, 1.86)	1.59 (0.98, 2.59)
pN+ and pT3/4	0.48 (0.28, 0.83)	1.07 (0.77, 1.48)	0.51 (0.23, 1.15)

* The final Cox regression model was used to estimate the hazard ratios. ^†^ The effect of CA27.29 after chemotherapy on disease-free survival varied between patient subgroups defined by pN and pT. ^‡^ Hazard ratios were adjusted for age, BMI, grading, ER, PR, HER2neu, and CA27.29 after chemotherapy. CA27.29 before chemotherapy was used as a nonlinear continuous predictor. It was evaluated in the first decile (“low”—i.e., 8.9 U/mL), at the median (“medium”—i.e., 17.4 U/mL), and in the ninth decile (“high”—i.e., 28.6 U/mL).

**Table 3:** Predicted 5-Year Disease-Free Survival Rates Relative to Patient Subgroups *.

Characteristic	5-Year Survival Rate (95% Confidence Intervals)		
	Low ^†^ CA27.29 before Chemotherapy	Medium CA27.29 before Chemotherapy	High CA27.29 before Chemotherapy
Age ^‡^			
Low	0.89 (0.85, 0.93)	0.89 (0.86, 0.92)	0.84 (0.79, 0.89)
Medium	0.90 (0.87, 0.94)	0.90 (0.87, 0.92)	0.85 (0.81, 0.89)
High	0.91 (0.88, 0.94)	0.91 (0.88, 0.93)	0.86 (0.82, 0.91)
BMI			
Low	0.90 (0.87, 0.94)	0.90 (0.87, 0.93)	0.85 (0.81, 0.90)
Medium	0.90 (0.87, 0.94)	0.90 (0.87, 0.92)	0.85 (0.81, 0.89)
High	0.90 (0.87, 0.94)	0.90 (0.87, 0.92)	0.85 (0.80, 0.90)
pT			
pT1	0.94 (0.92, 0.97)	0.93 (0.91, 0.95)	0.91 (0.87, 0.94)
pT2	0.90 (0.87, 0.94)	0.90 (0.87, 0.92)	0.85 (0.81, 0.89)
pT3/4	0.73 (0.61, 0.87)	0.86 (0.80, 0.92)	0.85 (0.78, 0.93)
pN			
pN0	0.96 (0.94, 0.98)	0.95 (0.94, 0.97)	0.95 (0.93, 0.98)
pN+	0.90 (0.87, 0.94)	0.90 (0.87, 0.92)	0.85 (0.81, 0.89)
Histology			
Ductal	0.90 (0.87, 0.94)	0.90 (0.87, 0.92)	0.85 (0.81, 0.89)
Lobular	0.88 (0.83, 0.93)	0.87 (0.83, 0.91)	0.81 (0.75, 0.88)
Other	0.93 (0.88, 0.97)	0.92 (0.88, 0.96)	0.89 (0.83, 0.95)
Grading			
G1	0.94 (0.92, 0.97)	0.94 (0.92, 0.96)	0.91 (0.88, 0.95)
G2	0.90 (0.87, 0.94)	0.90 (0.87, 0.92)	0.85 (0.81, 0.89)
G3	0.84 (0.78, 0.89)	0.83 (0.79, 0.87)	0.75 (0.69, 0.83)
ER			
ER−	0.86 (0.80, 0.92)	0.86 (0.81, 0.91)	0.79 (0.72, 0.87)
ER+	0.90 (0.87, 0.94)	0.90 (0.87, 0.92)	0.85 (0.81, 0.89)
PR			
PR−	0.84 (0.78, 0.91)	0.84 (0.79, 0.89)	0.76 (0.69, 0.85)
PR+	0.90 (0.87, 0.94)	0.90 (0.87, 0.92)	0.85 (0.81, 0.89)
HER2			
HER2−	0.90 (0.87, 0.94)	0.90 (0.87, 0.92)	0.85 (0.81, 0.89)
HER2+	0.93 (0.90, 0.96)	0.93 (0.90, 0.95)	0.89 (0.85, 0.93)
CA27.29 after chemotherapy			
Low	0.90 (0.86, 0.93)	0.89 (0.86, 0.92)	0.84 (0.79, 0.90)
Medium	0.90 (0.87, 0.94)	0.90 (0.87, 0.92)	0.85 (0.81, 0.89)
High	0.91 (0.87, 0.95)	0.91 (0.88, 0.93)	0.86 (0.82, 0.90)

* The final Cox regression model was used to estimate survival rates. Survival rates were estimated for a fictive patient belonging to a specific subgroup but is average with regard to all other characteristics. An “average patient” is considered to be a patient of median age, median BMI, and median CA27.29 after chemotherapy, with the most frequent characteristics (pT2, ductal, G2, ER+, PR+, HER2−). ^†^ CA27.29 before chemotherapy was used as a nonlinear continuous predictor. It was evaluated in the first decile (“low”—i.e., 8.9 U/mL), at the median (“medium”—i.e., 17.4 U/mL), and in the ninth decile (“high”—i.e., 28.6 U/mL). ^‡^ Age, BMI, and CA27.29 after chemotherapy were used as linear predictors. They were evaluated in the first decile (“low”—i.e., 39 years, 20.6 kg/m^2^, or 11.7 U/mL), at the median (“medium”—i.e., 53 years and 25.8 kg/m^2^, or 21.6 U/mL), and in the ninth decile (“high”—i.e., 67 years, 32.9 kg/m^2^, or 37.0 U/mL).

## Data Availability

The datasets used and analyzed during the current study are available from the corresponding author on reasonable request. The data are not publicly available due to ethical restrictions.

## Supplementary Materials

The following supporting information can be downloaded at: https://www.mdpi.com/article/10.3390/cancers14071721/s1, Figure S1: Distribution of the predicted 5-year disease-free survival probability (0–100%) in the study population (*n* = 2687); Table S1: Full Inclusion and Exclusion Criteria; Table S2: Performance of Cox Regression Models [23,24,25,26,27,28].

## Abbreviations

AE	Adverse event
ANC	Absolute neutrophil count
BC	Breast cancer
BMI	Body mass index
CI	Confidence interval
CMF	Cyclophosphamide, methotrexate and fluorouracil
CTC	Circulating tumor cells
DFS	Disease-free survival
Doc	Docetaxel
ER	Estrogen receptor
FEC	5-fluoroucacil, epirubicin and cyclophosphamide
5-FU	5-fluorouracil
G-CSF	Granulocyte-colony stimulating factor
Gem	Gemcitabine
HER2	Human epidermal growth factor receptor 2
HR	Hazard ratio
HRS	Hormone receptor status
MAPK	Mitogen-activated protein kinase
MUC1-C	Carboxy-terminal transmembrane subunit
MUC1-N	Amino-terminal extracellular subunit
NFκB	Nuclear factor kappa B
OS	Overall survival
PI3K	Phosphatidylinositol-3′-kinase
PR	Progesterone receptor
SGPT	Serum glutamate pyruvate transaminase
