# Supplementary material for: MUC1 (CA27.29) before and after Chemotherapy and Prognosis in High-Risk Early Breast Cancer Patients
Source: Cancers (Basel). 2022 Mar 28;14(7):1721. doi: 10.3390/cancers14071721 (PMC8997086; doi:10.3390/cancers14071721)
Supplement: Supplementary file 1 [file cancers-14-01721-s001.zip › cancers-1588195-supplementary.pdf]

Supplementary Materials: MUC1 (CA27.29) before and after Chemotherapy and Prognosis in High-Risk Early Breast Cancer Patients

Hanna Huebner, Lothar Häberle, Volkmar Müller, Iris Schrader, Ralf Lorenz, Helmut Forstbauer, Visnja Fink, Fabienne Schochter, Inga Bekes, Sven Mahner, Julia Jückstock, Naiba Nabieva, Andreas Schneeweiss, Hans Tesch, Sara Y. Brucker, Jens-Uwe Blohme, Tanja N. Fehm, Georg Heinrich, Mahdi Rezai, Matthias W. Beckmann, Peter A. Fasching, Wolfgang Janni and Brigitte Rack

Supplementary Methods

Treatments within SUCCESS A trial

In the SUCCESS-A open-label phase 3 trial, patients were randomly assigned at a ratio of 1:1 to either three cycles of fluorouracil, epirubicin, and cyclophosphamide (500/100/500 mg/m^2, FEC) followed by three cycles of docetaxel (100 mg/mg^2) every 3 weeks (q3w); or three cycles of FEC (at the same dosage and schedule as in the FEC→Doc randomization arm), followed by three cycles of gemcitabine (1,000 mg/m^2 d1,8) and docetaxel (75 mg/m^2) q3w. After chemotherapy, the patients were again randomized (in a 2 × 2 factorial design) to treatment with 2 vs. 5 years of zoledronate. Antihormonal therapy and anti-HER2 treatment were prespecified in the protocol: premenopausal hormone receptor–positive women received tamoxifen alone for 5 years, or in combination with goserelin for 2 years (patients younger than 40 years). Postmenopausal patients were treated with tamoxifen for 2 years, followed by anastrozole for 3 years. HER2-positive patients received trastuzumab for 1 year after completion of the chemotherapy. Surgery and radiotherapy had to be performed in accordance with national guidelines.

Statistical Methods

The primary objective was to study whether information about CA27.29 before and after chemotherapy improves the ability to predict disease-free survival for the patient, in addition to other well-known predictors. For this purpose, Cox regression analyses were performed as described below.

A mixed-effects Cox regression model (the "basic model") was fitted with disease-free survival as outcome, study center as random effect, and the following predictors as fixed effects: age at diagnosis (continuous), body mass index (BMI, continuous), pT (ordinal, pT1 to pT4), grading (ordinal, 1 to 3), lymph-node status (categorical, pN0 versus pN+), and PR (positive vs. negative), HER2 (positive vs. negative). ER (positive vs. negative) was used as a stratification factor rather than a predictor, because the proportional hazards assumption was not met for ER. Missing predictor values were imputed as described in Salmen et al.[1] Continuous predictors were used as natural cubic spline functions to describe nonlinear effects.[2] The number of degrees of freedom (1 to 3) for each predictor was determined as done recently in Salmen et al.[1] The variable "study center" was incorporated into the model as a random effect rather than a fixed effect or stratification variable, because of the large number of centers participating, which may diminish the power.[3] The proportional hazards assumptions were checked using the Grambsch and Therneau method.[4]

Next, an extended Cox model (the "full model") was fitted containing the predictors from the basic model, CA27.29 before chemotherapy (continuous), CA27.29 after chemotherapy (continuous), and interactions among these CA27.29 predictors by pT, pN, histology, HER2 and grading. Both CA27.29 predictors were used as cubic spline functions with three degrees of freedom to describe possible nonlinear relationships with the outcome. CA27.29 values above 50 U/mL were truncated.

The basic and the full model were compared using the likelihood ratio test. A significant test result means that CA27.29 influenced progression-free survival in addition to

the well-known predictors, either across all patients or at least within one of the subgroups defined by the interaction terms considered. In case of a nonsignificant result, no further analyses were carried out, in order to avoid false-positive results. However, if the *P* value was significant, the interaction model was compared with a reduced Cox model, the basic model with CA27.29 predictors added, but without the interaction terms (the "reduced model"), using the likelihood ratio test again.

In case of significance, the full Cox model was simplified. The complexity of the continuous predictors was optimized as done before in Salmen et al.,[1] and a backward stepwise selection procedure in which the predictors in the reduced model were kept was carried out to identify relevant interaction terms (the "final model"). Subgroup-specific HRs for CA27.29 before and after chemotherapy adjusted for the other predictors were calculated, using the final model. In the case of a nonsignificant result, adjusted overall HRs for both CA27.29 predictors were calculated, using the reduced model. The final model or, depending on the case, the reduced model was used for predicting 5-year survival rates. Interesting findings were illustrated using Kaplan–Meier curves.

Since significance in the predictors does not necessarily imply that these predictors will provide a basis for an effective prediction rule for individual patients,[5] the ability of the predictors to discriminate between patients with disease recurrence within 2 or 5 years, respectively, and those without was assessed using the time-dependent area under the curve (AUC) for survival analyses.[6] Large AUC values might justify a search for thresholds for classifying patients as "high risk" or "low risk" with regard to survival. To obtain reliable results, each AUC value was estimated by 10-fold cross-validation with 20 repetitions. The complete model-building process (i.e., the determination of cubic spline functions and variable selection) was carried out on each training set, resulting in several Cox models (one model per set), which were then used to calculate the AUC on the corresponding validation datasets that were not used for model building. The average of all these AUCs was taken as a measure (cross-validated AUC). To assess model overfitting, the AUC was also calculated with the complete dataset using a model fitted on the (same) complete dataset. Differences between this apparent AUC and the cross-validated AUC indicate overfitting.

To further analyze the predictive ability of CA27.29 before and after chemotherapy, another two Cox regression models were fitted, each with the basic predictors and either CA27.29 before or CA27.29 after chemotherapy. Model performances were assessed as described above.

All of the tests were two-sided, and a *P* value of < 0.05 was regarded as statistically significant. Calculations were carried out using the R system for statistical computing (version 3.0.1; R Development Core Team, Vienna, Austria, 2013).

References

1. Salmen, J.; Neugebauer, J.; Fasching, P.A.; Haeberle, L.; Huober, J.; Wockel, A.; Rauh, C.; Schuetz, F.; Weissenbacher, T.; Kost, B.; et al. Pooled analysis of the prognostic relevance of progesterone receptor status in five German cohort studies. *Breast Cancer Res. Treat.* **2014**, *148*, 143–151. https://doi.org/10.1007/s10549-014-3130-4.
2. Hastie, T.; Tibshirani, R. Generalized additive models for medical research. *Stat. Methods Med. Res.* **1995**, *4*, 187–196.
3. Ripatti, S.; Palmgren, J. Estimation of multivariate frailty models using penalized partial likelihood. *Biometrics* **2000**, *56*, 1016–1022.
4. Grambsch, P.M.; Therneau, T.M. Proportional Hazards Tests and Diagnostics Based on Weighted Residuals. *Biometrika* **1994**, *81*, 515–526.
5. Ware, J.H. The limitations of risk factors as prognostic tools. *N. Engl. J. Med.* **2006**, *355*, 2615–2617. https://doi.org/10.1056/NEJMp068249.
6. Uno, H.; Cai, T.X.; Tian, L.; Wei, L.J. Evaluating prediction rules for t-year survivors with censored regression models. *J. Am. Stat. Assoc.* **2007**, *102*, 527–537. https://doi.org/10.1198/016214507000000149.

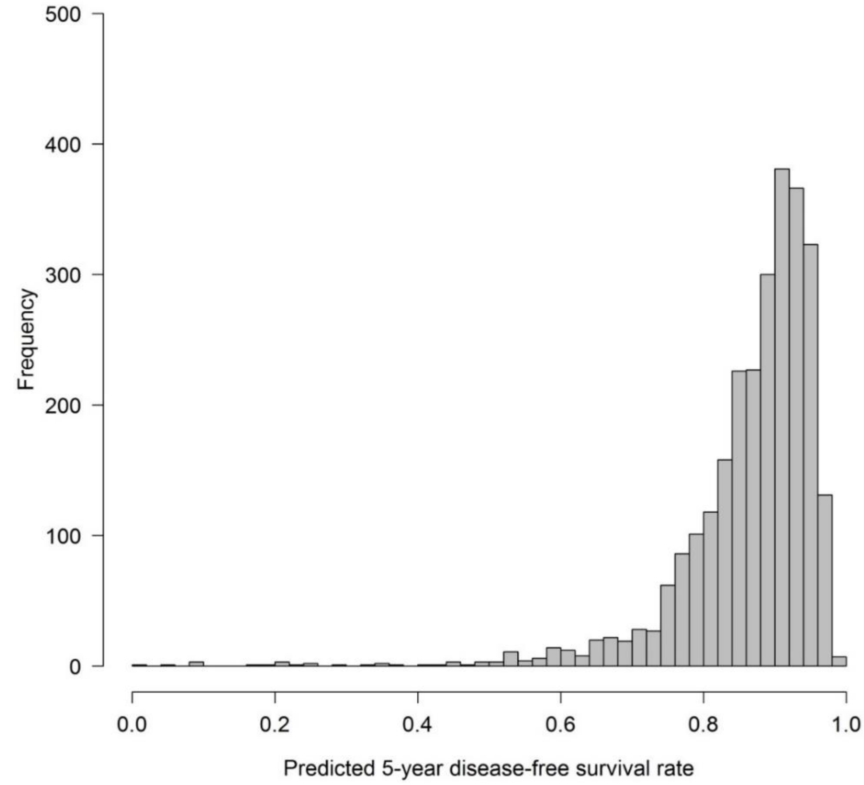

Figure S1. Distribution of the predicted 5-year disease-free survival probability (0–100%) in the study population (n = 2,687). The bars show how many patients (in percentages) had a predicted probability between 0% and 2%, between 2% and 4%, etc. Half of all patients had a predicted probability between 82.6% and 92.5% (interquartile range). The median probability was 88.6%.

Table S1. Full Inclusion and Exclusion Criteria.

	Inclusion criteria
	**Patients may be included in the study only if they meet all the following criteria:**
**1**	Primary epithelial invasive carcinoma of the breast pT1–4, pM0
2	Histopathological confirmation of axillary lymph-node metastases (pN1–3) or high-risk pN0/NX, defined as: "pT ≥ 2 or histopathological grade 3 or age ≤ 35 or negative hormone receptor status"
3	Complete resection of the primary tumor, with resection margins free of invasive carcinoma, no more than 6 weeks previously
4	Females ≥ 18 years of age
5	Performance status ≤ 2 on the Eastern Cooperative Oncology Group (ECOG) scale
6	Adequate bone marrow reserve: leukocytes ≥ 3.0×10^9/L and platelets ≥ 100×10^9/L
7	Bilirubin within 1-fold of the reference laboratory's normal range, ASAT (SGOT), ALAT (SGPT) and AP within 1.5-fold of the reference laboratory's normal range for patients
8	Intention to attend regular follow-up visits for the duration of the study
9	Ability to understand the nature of the study and to provide written informed consent
	Exclusion criteria
	Patients will be excluded from the study for any of the following reasons:
10	Inflammatory breast cancer
11	Previous or concomitant cytotoxic or other systemic antineoplastic treatment that is not part of or not allowed in this study
12	History of treatment or disease affecting bone metabolism (e.g., Paget's disease, primary hyperparathyroidism)
13	Prior treatment with bisphosphonates within the previous 6 months
14	Severe renal insufficiency as evidenced by creatinine clearance (CrCl) < 30 mL/min, as calculated using the Cockcroft–Gault formula: $CrCl = \frac{140 - age(years) * weight\ (kg) * 0.85}{72 * serum\ creatinine\ (\frac{mg}{dl})}$
15	Second primary malignancy (except in situ carcinoma of the cervix or adequately treated basal cell carcinoma of the skin)
16	Cardiomyopathy with impaired ventricular function (New York Heart Association > II), cardiac arrhythmias influencing left ventricular ejection fraction and requiring medication, history of myocardial infarction or angina pectoris within the previous 6 months, or arterial hypertension not controlled by medication
17	Any known hypersensitivity against docetaxel, epirubicin, cyclophosphamide, fluorouracil, gemcitabine, or any other medication included in the study protocol
18	Use of any investigational agent within 3 weeks prior to inclusion
19	Patients in pregnancy or breastfeeding (in premenopausal women contraception has to be ensured: intrauterine devices, surgical sterilization methods, or — in hormone-insensitive tumors only — oral, subcutaneous or transvaginal hormonal, non–estrogen-containing contraceptives)
20	Current active dental problems, including infection of the teeth or jaw (maxilla or mandible); dental or fixture trauma, or a current or prior diagnosis of osteonecrosis of the jaw (ONJ), of exposed bone in the mouth, or of slow healing after dental procedures
21	Recent (within 6 weeks) or planned dental or jaw surgery (e.g., extraction, implants)

Table S2. Performance of Cox Regression Models. Showing Apparent * and Cross-Validated † AUC Values after 2 Years and 5 Years of Follow-Up.

**Model**	**AUC at Year 2**		**AUC at Year 5**	
	**Apparent**	**Cross-Validated**	**Apparent**	**Cross-Validated**
Basic model	0.710	0.690	0.700	0.675
Basic + CA27.29 before chemotherapy	0.726	0.703	0.717	0.690
Basic + CA27.29 after chemotherapy	0.708	0.689	0.700	0.675
Basic + both CA27.29 (reduced model)	0.727	0.704	0.718	0.692
Full model	0.821	0.743	0.780	0.706
Final model	0.737	0.702	0.742	0.700

AUC, area under the curve. * The complete dataset was used for fitting the Cox regression models and calculating the AUC afterwards. † The complete dataset was repeatedly split into training and validation datasets. Model fitting was carried out on the training data, whereas AUC calculation was carried out on the validation data.
